# Transcriptional Regulation of Glucose Sensors in Pancreatic β-Cells and Liver: An Update

**DOI:** 10.3390/s100505031

**Published:** 2010-05-19

**Authors:** Jin-Sik Bae, Tae-Hyun Kim, Mi-Young Kim, Joo-Man Park, Yong-Ho Ahn

**Affiliations:** 1 Department of Biochemistry and Molecular Biology, Yonsei University College of Medicine, Seoul 120-752, Korea; E-Mails: baejinsik@yuhs.ac (J.-S.B.); taehyun@yuhs.ac (T.-H.K.); theresakim@yuhs.ac (M.-Y.K.); jjumans@yuhs.ac (J.-M.P.); 2 Center for Chronic Metabolic Disease Research, Yonsei University College of Medicine, Seoul, Korea; 3 Brain Korea 21 Project for Medical Sciences, Yonsei University College of Medicine, Seoul, Korea

**Keywords:** glucose sensor, solute carrier family 2 (*SLC2A2*), glucokinase (*GCK*), transcription, liver, pancreatic β-cell

## Abstract

Pancreatic β-cells and the liver play a key role in glucose homeostasis. After a meal or in a state of hyperglycemia, glucose is transported into the β-cells or hepatocytes where it is metabolized. In the β-cells, glucose is metabolized to increase the ATP:ADP ratio, resulting in the secretion of insulin stored in the vesicle. In the hepatocytes, glucose is metabolized to CO_2_, fatty acids or stored as glycogen. In these cells, solute carrier family 2 (*SLC2A2*) and glucokinase play a key role in sensing and uptaking glucose. Dysfunction of these proteins results in the hyperglycemia which is one of the characteristics of type 2 diabetes mellitus (T2DM). Thus, studies on the molecular mechanisms of their transcriptional regulations are important in understanding pathogenesis and combating T2DM. In this paper, we will review a recent update on the progress of gene regulation of glucose sensors in the liver and β-cells.

## 1. Introduction

Glucose is one of the most important molecules that acts as a basic fuel for energy source and a substrate for intermediary metabolism as well. Because of its essential role in the metabolism, most cells have evolved to have an apparatus to sense and transport extracellular glucose into the cells. The glucose sensing in mammalian cells is regulated by both direct and indirect pathways. In the postprandial state, temporarily increased glucose has to be disposed of to prevent the cells from gluco-toxicity. After meal, glucose in the blood is absorbed near the portal vein, and metabolized in the liver and pancreas [1].

Blood glucose is transported into the liver and β-cells of pancreas through solute carrier family 2 (SLC2A2, also known as GLUT2) and immediately phosphorylated by glucokinase present in the liver (LGCK) or β-cells (βGCK) which acts as a glucose sensor. Glucose-6-phosphate in the hepatocytes undergoes glycolysis, glycogenesis, pentose phosphate pathway, or hexosamine biosynthetic pathway depending on the metabolic needs. Both SLC2A2 and GCK have high *K*_m_ values and high capacity and thus are able to sense and transport glucose into hepatocytes or β-cells in proportion to the blood glucose level [2].

In the β-cells, glucose is metabolized and thereby increases intracellular the ATP:ADP ratio which causes suppression of ATP-sensitive K^+^ channel and triggers insulin secretion [3–5]. In addition, the gene expression of insulin is stimulated by glucose and is subjected to control at the transcriptional level.

Most of the type 2 diabetes mellitus (T2DM)-associated genes are mainly involved both in β-cell function and peripheral insulin sensitivity. Mutations in the *GCK* gene are associated with maturity onset diabetes of the young (MODY), a subtype of diabetes characterized by monogenic autosomal dominant transmission, early age of onset (typically less than 25 years of age) and primary defects in β-cell function. MODY are also associated with mutations in the genes encoding transcription factors like, hepatic nuclear factor 4 alpha (*HNF4A*), HNF1 homeobox A (*HNF1A*), pancreatic and duodenal homeobox 1 (*PDX1*), HNF1 homeobox B (*HNF1B*) and neurogenic differentiation 1 (*NEUROD1*) [6–8]. Furthermore, these transcription factors are known to be involved in the regulation of tissue-specific expression of *SLC2A2* and/or *GCK* genes [9]. Dysfunctional mutation in *SLC2A2* gene is also found in one patient with T2DM [10].

The gene expression of *SLC2A2* and *GCK* is affected by metabolic conditions and are also tissue-specific. SLC2A2 is primarily expressed in the liver and β-cells [11,12] and its gene expression is affected by the blood glucose and insulin [13,14]. In diabetic animal models, *SLC2A2* mRNA level is increased in the liver [15], whereas it is decreased in β-cells [16,17]. GCK is expressed mainly in the mammalian liver and β-cells, with two alternative promoters that govern tissue-specific expression [18–21]. The β*GCK* promoter (upstream promoter) is regulated by glucose, whereas the L*GCK* promoter (downstream promoter) is regulated by insulin and glucagon [21].

In this review, we will focus on an update on the transcriptional regulation of *SLC2A2* and *GCK* genes in the liver and β-cells. Studying the molecular mechanisms in relation to T2DM will help understand its pathogenesis and find potential drug targets for the development of therapeutic drugs.

## 2. Transcriptional Regulation of *SLC2A2* in the Liver and β-Cells of Pancreas

**Figure 1.**
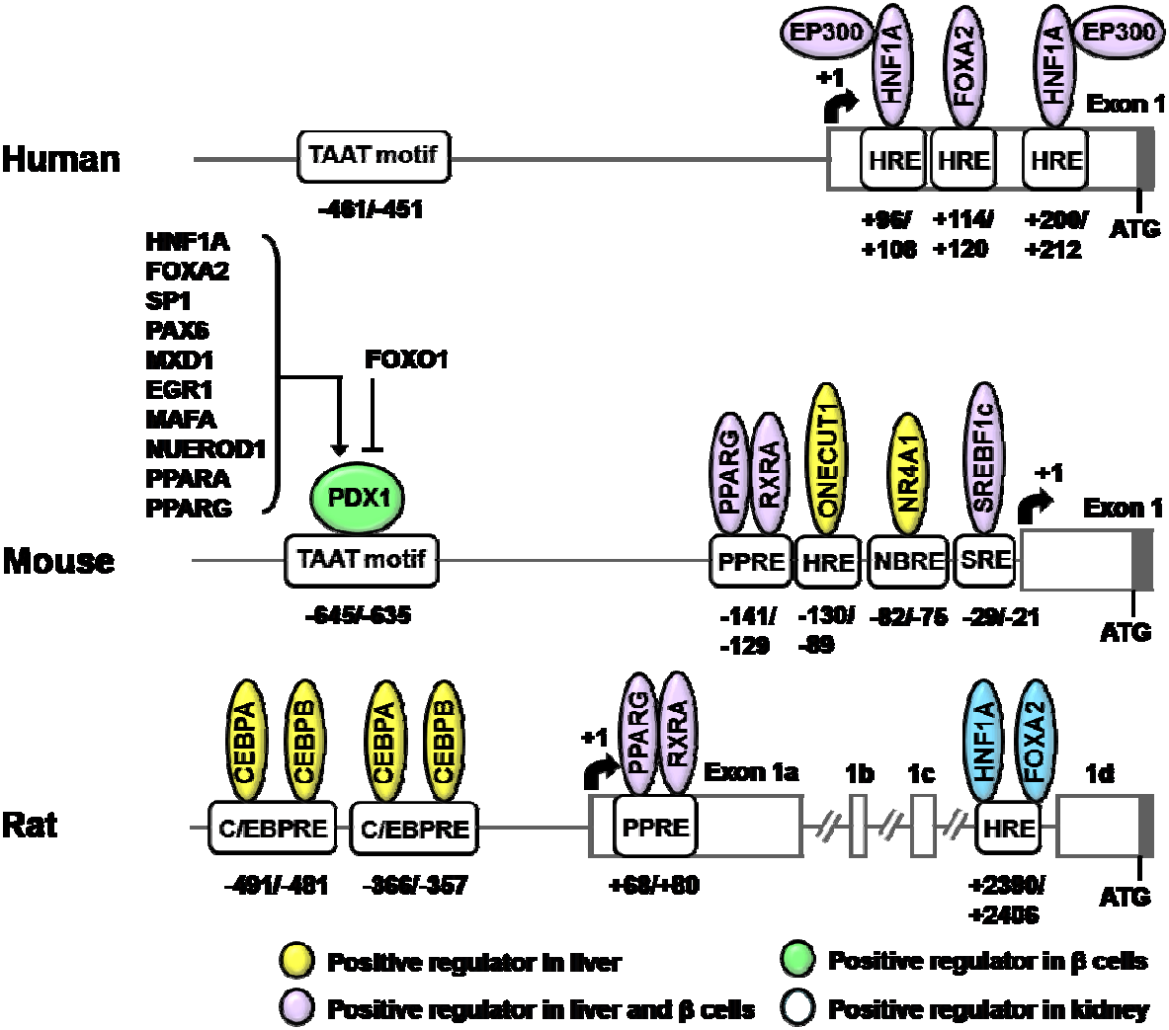
Schematics of transcriptional regulatory elements on the *SLC2A2* gene promoter. *Abbreviations*: HNF1A, HNF1 homeobox A; EP300, E1A binding protein p300; FOXA2, forkhead box A2 (also known as HNF3B); PDX1, pancreatic and duodenal homeobox 1; SP1, Sp1 transcription factor; PAX6, paired box 6; MXD1, MAX dimerization protein 1; EGR1, early growth response 1; MAFA, v-maf musculoaponeurotic fibrosarcoma oncogene homolog A; NEUROD1, neurogenic differentiation 1; PPARA, peroxisome proliferator-activated receptor alpha; PPARG, peroxisome proliferator-activated receptor gamma; FOXO1, forkhead box O1; RXRA, retinoic X receptor alpha; ONECUT1, one cut homeobox 1 (also known as HNF6); NR4A1, nuclear receptor subfamily 4, group A, member 1; SREBF1c, sterol regulatory element binding transcription factor 1c; CEBPA, CCAAT/enhancer binding protein (C/EBP) alpha; CEBPB, CCAAT/enhancer binding protein (C/EBP) beta; HRE, HNF response element; PPRE, PPAR response element; NBRE, nerve growth factor I-B response element; SRE, SREBF response element; C/EBPRE, CEBP response element. +1, transcription start site.

Since the cloning of the promoter regions of *SLC2A2* gene, numerous studies on the transcriptional regulation have been performed (for a review see [9]). Unlike those of human or mouse *SLC2A2* genes, rat *Slc2a2* promoter contains three noncoding exons (exons 1A, 1B, and 1C; Figure 1) [22]. As shown, several HNFs are involved in transcriptional regulation of *SLC2A2* genes.

HNF1A is an essential transcription factor for the expression of *Slc2a2* gene in β-cells. In transgenic mice over-expressing a dominant negative form of HNF1A, the expression of *Slc2a2* gene is decreased in the pancreatic islets. In *Hnf1a* knockout mice, the expression of *Slc2a2* gene was decreased in the pancreatic islets, but not affected in the liver [23–25].

Both HNF1A and forkhead box A2 (FOXA2, also known as HNF3B) are responsible for the tissue-specific expression of the human *SLC2A2* gene. These factors synergistically increase the promoter activity of human *SLC2A2* gene in NIH-3T3 cells. Binding of HNF1A and FOXA2 to +96/+108 and +114/+120 bp region of human *SLC2A2* promoter was identified and these binding sites were well conserved in the mouse and rat gene [26]. HNF1A and FOXA2 also upregulate *Slc2a2* mRNA in the kidney of diabetic rats [27]. Another HNF1A binding site (+200/+212 bp) was found in the promoter of human *SLC2A2* gene. A mutation study revealed that the +200/+212 bp site is more important than the +96/+108 bp one in HNF1A-induced *SLC2A2* gene expression. Moreover, E1A binding protein p300 (EP300) potentiates activity of the human *SLC2A2* promoter by interacting with the transactivation domain of HNF1A [28].

In transgenic mice or adenoviral transduction of recombinant *Foxa2* (Ad*Foxa2*), *Slc2a2* mRNA level was decreased in the liver [29, 30], presumably because FOXA2 represses one cut homeobox 1 (*Onecut1*, also known as *Hnf6*) gene expression, which is a positive regulator of *Slc2a2* gene in the mouse liver [31]. However, mRNA levels of *Slc2a2* and *Onecut1* were not altered in liver specific *Foxa2* knockout mice [32]. In addition, *Slc2a2* gene expression was not altered in β-cell specific *Foxa2* knockout mice [33]. These reports suggest that transactivating effect of FOXA2 on *Slc2a2* promoter may be weak or absent in mouse liver and β-cells.

FOXA3 (also known as HNF3G) is known to act as a positive regulator of the *Slc2a2* gene in the liver, although upregulation of the gene was not observed in β-cells [34]. HNF4A is also known to activate *SLC2A2* gene expression in embryonic stem cells [35] and β-cells [36].

PDX1 plays a key role in the development of pancreas by orchestrating gene regulation in β-cells [37] and is known to upregulate *Slc2a2* gene expression through TAAT motif in the *Slc2a2* promoter [38]. *Slc2a2* gene expression in the β-cell specific *Pdx1* knockout [39,40] and *Pdx1* heterozygote mice [41] is dramatically reduced when compared to that of wild type mice.

Although PDX1 was shown to bind *in vitro* to the promoter region of β-cell specific genes, including *Slc2a2* (EMSA data), chromatin immunoprecipitation (ChIP) assays indicated that PDX1 did not bind to the promoter of *Slc2a2* gene in the β-TC3 cells. These results suggest that selectivity of PDX1 may depend on the cell type specific chromatin structures and/or the presence of interacting proteins [42]. Indeed, PDX1 of which binding to *Slc2a2* promoter is reinforced by high-mobility group N 3 (HMGN3), a chromatin binding protein that is highly expressed in β-cells [43].

Since various transcription factors, like HNF1A, FOXA2, Sp1 transcription factor (SP1) [44], paired box 6 (PAX6) [45], MAX dimerization protein 1 (MXD1) [46], early growth response-1 (EGR1) [47,48], v-maf musculoaponeurotic fibrosarcoma oncogene homolog A (MAFA) [49], neurogenic differentiation 1 (NEUROD1) [50], peroxisome proliferator–activated receptor alpha (PPARA) [51] and peroxisome proliferator–activated receptor gamma (PPARG) [52], were known to be positive-regulators of *PDX1* gene, it was speculated that many of these transcription factors may indirectly affect gene expression of *SLC2A2* through PDX1. *Slc2a2* mRNA level was decreased in INS832/13 cells in which forkhead transcription factor FOXO1 is overexpressed [53]. Since FOXO1 binds to the *PDX1* promoter and inhibits FOXA2-induced PDX1 expression, the effect of FOXO1-suppression of *Slc2a2* gene expression might occur in an indirect way [54].

V-maf musculoaponeurotic fibrosarcoma oncogene homolog B (MAFB) is a critical transcription factor for β-cell differentiation, although its expression was not observed in adult pancreas β-cells [55–57]. *Slc2a2* and *Pdx1* gene expression is reduced in the embryonic pancreas of *Mafb* knockout mice and MAFB binds to the promoter of *Slc2a2* gene *in vivo* [57]. Because PDX1 is a positive regulator of *Slc2a2* gene expression, *Slc2a2* gene expression may be regulated by MAFB either directly or indirectly. Furthermore, *Slc2a2* expression is decreased in *Mafa*-deficient mice [58]. However, MAF response element(s) in the *Slc2a2* promoter has not been identified.

PPARG directly activates *SLC2A2* gene expression in the liver and β-cells [59–61]. Rosiglitazone increased *SLC2A2* mRNA level in the primary cultured hepatocytes, Alexander [59] and INS-1 cells [62]. Troglitazone also increased *Slc2a2* mRNA level in primary cultured islets from rats [52,60]. In addition, decreased *Slc2a2* gene expression in the islets of *db*/*db* mouse was restored by pioglitazone treatment [63, 64]. RNAi-suppression of *Pparg* in INS-1 cells caused reduction in *Slc2a2* mRNA levels [52]. The functional PPAR response elements (PPREs) have been identified in the promoters of rat [60] and mouse [59] *Slc2a2* gene. Therefore, thiazolidinediones (TZDs) may contribute to the transport glucose into the liver or β-cell by upregulating *SLC2A2* gene [61]. PPARA also upregulates the *Slc2a2* gene expression in the β-cells [51,65]. *Ppara* null mice showed low level of *Slc2a2* mRNA in the pancreas [66].

Glucose increased the *Slc2a2* gene expression in the liver and β-cells, both *in vivo* and *in vitro* [14,67–70]. Recently, we have identified a sterol regulatory element binding transcription factor 1 (SREBF1) response element (SRE) in the promoter of mouse *Slc2a2* gene, which is responsive to glucose in primary cultured hepatocytes [69]. Furthermore, glucose-induction of the *Slc2a2* gene expression in pancreatic islets was not found in *Srebf1* knockout mice [70].

Also, cyclic adenosine monophosphate (cAMP) prevents the glucose-mediated stimulation of *Slc2a2* gene expression in hepatocytes. The −312/+49 bp region of the mouse *Slc2a2* promoter is responsible for cAMP responsiveness [71]. However, functional cAMP response element(s) within this region has not been identified.

An orphan nuclear receptor NR4A1 (nuclear receptor subfamily 4, group A, member 1) binds to nerve growth factor I-B response element (NBRE) in mouse *Slc2a2* promoter (−82/–75 bp) and increases mRNA level in primary cultured hepatocytes and liver. Furthermore, *Slc2a2* mRNA induced by NR4A1 is synergistically enhanced by PPARG coactivator 1 alpha (PPARGC1A) [72]. Because NR4A1 expression is highly regulated by the cAMP axis in the liver [72], these results are not consistent with the previous report that cAMP decreases the promoter activity of the *Slc2a2* gene [71]. Further studies are needed to elucidate these contradictory results.

Insulin plays a negative role in *Slc2a2* gene expression in the liver [14]. Since insulin-FOXO1 pathway is responsible for the negative role in insulin-mediated gene expression [73], it is tempting to speculate that FOXO1 may be a negative regulator of *Slc2a2* gene expression. However, there is no evidence that FOXO1 is involved in the gene expression of *Slc2a2* in the liver, although its negative role of FOXO1 in the *Slc2a2* gene expression was shown in β-cells.

CCAAT/enhancer binding protein (CEBP) is shown to activate rat *Slc2a2* promoter in HepG2 cells. The promoter has two CEBP consensus sequences binding CEBPA and CEBPB (Figure 1). These factors synergistically activate the promoter [74].

Kruppel-like factor 7 (KLF7) is known to reduce mRNA level of *SLC2A2* in HIT-T15 and HepG2 cells. Because KLF7 is shown to reduce *PDX1* gene expression in HIT-T15 cells [75], *SLC2A2* gene expression may be regulated by KLF7 either directly or indirectly.

Although tissue-specific transcriptional regulation is not absolutely consistent between human and mouse [76], *SLC2A2* gene expression in liver was activated by ONECUT1, FOXA3, PPARG, SREBF1c, NR4A1, CEBPA, CEBPB and KLF7. On the other hand, *SLC2A2* gene expression in β-cells was activated by HNF1A, HNF4A, PDX1, HMGN3, MAFA, MAFB, PPARA, PPARG and KLF7 and was suppressed by FOXO1. The gene expression or activity of these regulators in abnormal conditions like high-fat or high-carbohydrate diet and cellular stress may contribute to the etiology of T2DM.

## 3. Transcriptional Regulation of Glucokinase (GCK)

### 3.1. Beta Cell Glucokinase (βGCK)

GCK plays a critical role in maintaining the postprandial glucose level near 5 mM, which is achieved by glucose stimulated insulin secretion (GSIS) from β-cells and glucose metabolism in the liver [77]. βGCK is a primary determinant of blood glucose level because it senses glucose for GSIS.

Upregulation of β*Gck* gene expression by glucose is mediated by insulin. In this mechanism, insulin receptor B type, PI3K class 1a and p70 s6 kinase pathway are known to be involved in glucose-regulated β*Gck* transcription [78]. Furthermore, β*Gck* gene expression is increased when MIN6 cells were cultured at 30 mM glucose [79]. However, β*Gck* mRNA level is not changed in rats which are subjected to fasting/refeeding although L*Gck* gene expression is significantly increased [80]. Indeed, the 4 kb promoter reporter construct of the β*Gck* gene was not activated either by glucose (30 mM) or insulin (20 nM) [81]. These studies indicate that the role of glucose or insulin in the activation of βGCK may occur by stabilization rather than upregulation of β*Gck* gene expression in insulinoma cells [21].

**Figure 2.**
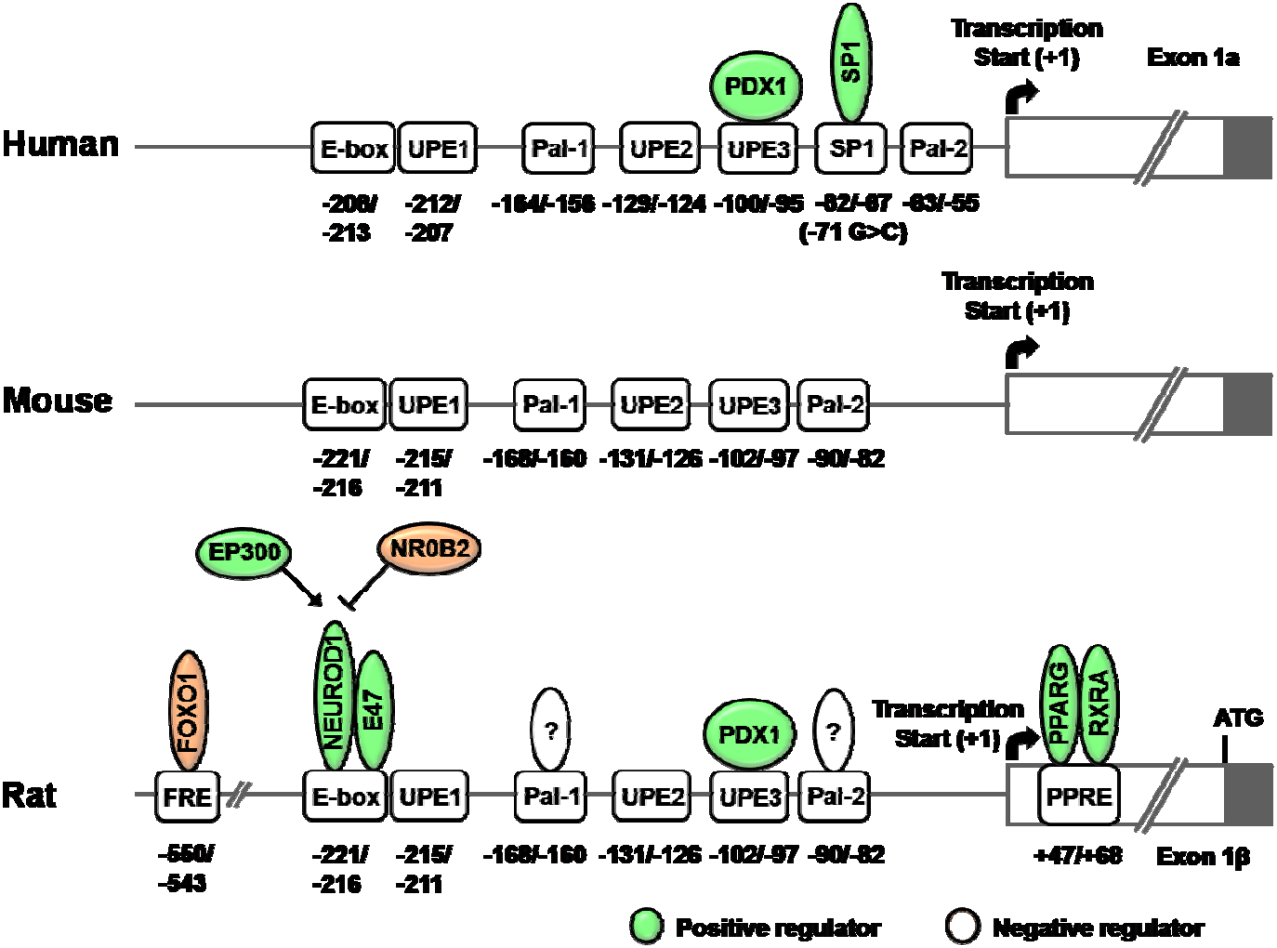
Schematics of transcriptional regulatory elements on the β*GCK* gene promoter. *Abbreviations*: PDX1, pancreatic and duodenal homeobox 1; SP1, Sp1 transcription factor; FOXO1, forkhead box O1; NEUROD1, neurogenic differentiation 1; E47, an immunoglobulin enhancer-binding factor; EP300, E1A binding protein p300; NR0B2, nuclear receptor subfamily 0, group B, member 2 (also known as SHP); PPARG, peroxisome proliferator-activated receptor gamma; RXRA, retinoic X receptor alpha; UPE, upstream promoter element; FRE, FOXO1 response element; PPRE, PPAR response element.

As shown in Figure 2, the gene expression of β*GCK* is regulated by various transcription factors. In its 5′-flanking region, there are three upstream promoter elements (UPEs) and two Pal motifs. Particulary, UPE-3 and Pal motifs are well conserved in the rat, mouse and human genes [19,82,83]. The Pal motifs consist of inverted repeats separated by 1 bp (TGGTCACCA). The promoter activity of β*Gck* gene was decreased by the introduction of mutation in the Pal motifs. These Pal motifs are pivotal determinants for the neural/neuroendocrine cell-specific expression of the *Gck* promoter [82,83].

UPEs have AT-rich sequence, which is known to be responsible for PDX1 activation. PDX1 is a master regulator for maintaining function and differentiation of β-cell. In the presence of glucose, PDX1 is phosphorylated and translocated into nucleus [84–86]. Expression of PDX1 increased the reporter activity of β*Gck* promoter in CHO cells. PDX1 binding site is conserved at UPE3 region in human β*GCK* promoter and may play an essential role for β-cell function [87]. However, β-cell specific disruption of *Pdx1* did not affect the expression of β*Gck* although *Slc2a2* expression was down-regulated [39]. The transcriptional role of PDX1 on the β*GCK* gene is not fully understood.

A basic helix-loop-helix (bHLH) transcription factor, NEUROD1 is known to bind β*Gck* promoter (−221/–216 bp; E-box) with E47 as a heterodimeric partner and transactivates the β*Gck* gene [88]. In addition, nuclear receptor subfamily 0, group B, member 2 (NR0B2, also known as SHP) interacts with NEUROD1 and represses the transcriptional activity of NEUROD1 by competing with coactivator EP300 [89]

PPARG/RXRA (retinoic X receptor alpha) binds to the promoter of rat β*Gck* gene of which binding element (PPRE) is located at +47/+68 bp. In addition, troglitazone increased the endogenous expression and enzyme activity of βGCK [24]. Knockdown of *Pparg* using siRNA resulted in a decrease in the mRNA level of *Pdx1*, *Gck, Slc2a2* and insulin II [52].

In addition, insulin-like growth factor 1 (IGF1) is known to induce β*Gck* gene expression by phosphorylating FOXO1. FOXO1 response element (FRE) is located at −550/–543 bp of rat β*Gck* promoter and FOXO1 binding to FRE is decreased by IGF1 *in vitro* [90].

Although PDX1, NEUROD1 and NK2 homeobox 2 (NKX2-2) bind to the region of −285/–5 bp in the mouse β*Gck* promoter, their respective response elements in the β*Gck* promoter have not been characterized [91]. Consistent with a role of NKX2-2 on the β*Gck* promoter, *Nkx2-2* knockout mice revealed a reduction in the β*Gck* mRNA level. In addition, NKX2-2 appeared to play an important role for the differentiation of β-cells [92].

### 3.2. Liver Glucokinase (LGCK)

The gene expression of L*Gck* is decreased in streptozotocin-induced diabetic rats and restored by insulin administration [93]. In addition, L*Gck* gene expression is increased by insulin and decreased by the glucagons-cAMP system in primary cultured hepatocytes [94]. Insulin-induction of L*Gck* gene expression is shown to be blocked by LY294002 or wortmannin, a PI3K inhibitor [95]. Furthermore, L*Gck* gene expression is inhibited by a dominant negative form of insulin receptor substrate 1 (IRS1) [96]. These studies support that insulin is a principal regulator of L*Gck* gene expression.

SREBF1c, one of the master regulators of lipogenesis, is dramatically induced by insulin [97,98]. Administration of recombinant adenovirus of *Srebf1c* to streptozotocin-induced diabetic mice restored L*Gck* and lipogenic enzymes normalizing blood glucose level despite that insulin is absent [99]. In addition, adenoviral expression of dominant negative form of SREBF1c in primary cultured hepatocytes decreased insulin-induction of L*Gck* gene, suggesting a direct participation of SREBF1c in the L*Gck* gene expression [100]. Furthermore, direct binding site of SREBF1c on rat L*Gck* promoter is identified [101]. However, L*Gck* gene expression is still increased by refeeding the *Srebf1c* knockout mice [102]. Moreover, L*Gck* gene expression is not changed even though *Srebf1c* was knockdown by siRNA although fatty acid synthase mRNA level is decreased. These studies suggest that SREBF1c is not likely to be a mediator of L*Gck* gene expression [103, 104]. Further studies are needed to answer these contradictory results.

HNF4A is known to be an important transcription factor for glucose and lipid homeostasis [35,105]. HNF4A increases the L*GCK* gene expression and its binding site HRE (HNF response element) is identified in human [106] and rat [107]. During the fasting period, L*GCK* transcription by HNF4A is repressed by FOXO1 which acts as a corepressor, whereas the suppression is restored by feeding where FOXO1 is phosphorylated and extruded to cytosol by insulin [106].

Hypoxia inducible factor 1 alpha subunit (HIF1A) also affects promoter activity of rat L*Gck* gene. HIF1A binding site is localized at −87/−80 bp region of the promoter. Both insulin [108] and hypoxia [21,109,110] upregulate L*Gck* gene expression by increasing HIF1A level and its DNA-binding activity. Transactivation by HIF1A was also enhanced by co-expression of HNF4A and EP300. Moreover, HIF1A interacts with HNF4A and each of these factors also interacts with EP300. It was suggested that synergy and cooperative interactions between HIF1A, HNF4A and EP300 might be necessary for insulin-stimulated L*Gck* expression [109].

Signal transducer and activator of transcription 5B (STAT5B) is known to be regulated by insulin [111,112]. STAT5B phosphorylated by insulin is translocated to the nucleus and increases its binding to the *cis*-acting elements, thereby increasing the transcription of target genes. Activated STAT5B by insulin increased L*GCK* gene expression [113,114]. In humans, the binding site of STAT5B is characterized at −1368/−1360 bp region, but the mouse STAT5 response element (STAT5RE) was unknown. The binding affinity of STAT5B to the human L*GCK* promoter is also increased by insulin and the activation by insulin occurs in a janus kinase (JAK)-independent manner. Thus, it is suggested that STAT5B plays an important role in the insulin-mediated upregulation of L*Gck* gene [113]. However, insulin is known to increase the transcription of L*Gck* in primary cultured hepatocytes although the tyrosine phosphorylation of STAT5 was not detectable [114].

During the fasting period, sirtuin 1 (SIRT1), an NAD^+^-dependent deacetylase, decreases the rat L*Gck* gene expression by deacetylating FOXO1, which results in an increase in binding of FOXO1 to FRE (−537/–529 bp) of its promoter. They also observed that resveratrol enhances interaction of between FOXO1 and HNF4A, causing a decrease in the binding affinity of HNF4A to the HRE [115].

Upstream stimulating factor 2 (USF2) is responsible for the regulation of L*Gck* gene expression by binding to the P2 element (−89/–81 bp), and thus, the transcription factor may be in part responsible for the glucose homeostasis [116].

TZDs are anti-diabetic drugs improving glucose utilization and insulin sensitivity. Troglitazone is a synthetic ligand of PPARG and is shown to upregulate L*Gck* gene expression. The PPRE is located at the −116/–104 bp of L*Gck* gene promoter [117].

Recently, liver X receptor alpha (LXRA, also known as NR1H3) was shown to upregulate L*Gck* gene expression by binding to the LXR response element (LXRE) (−52/–39 bp) in its promoter. In addition, LXRA increases L*Gck* gene expression by inducing SREBF1c and increasing transcriptional activity of PPARG. Furthermore, NR0B2 induced by LXRA plays a role in the fine-tuning the L*Gck* gene expression [118]. Because the binding site of LXRA and HNF4A seems to be overlapped [107], detailed studies are needed to elaborate their precise roles of these factors with regard to specific metabolic conditions.

In mice, ONECUT1 binding site in the L*Gck* gene promoter is localized at −7613/–7622 bp and −877/–868 bp, suggesting a possible link between *Onecut1* deficiency and development of T2DM. This study could explain why T2DM occurs in *Onecut1* knockout mice [119,120].

## 4. Effect of Promoter Polymorphisms on *SLC2A2* and *GCK* Transcription

Single nucleotide polymorphisms (SNPs) in the promoter regions can affect the binding of transcription factors regulating the transcription of genes. Some significant promoter SNPs of *SLC2A2* and *GCK* genes were reported in T2DM patients.

### 4.1. Promoter Polymorphisms in SLC2A2 Gene

Three common SNPs, −149C > A (rs5393), −122T > C (rs5394) and −44G > A (rs5396), in *SLC2A2* promoter were identified in a Danish population. These SNPs were not significantly different in the genotype frequency between T2DM patients and control subjects. In addition, clinical characteristics of T2DM were not significantly associated [121]. However, SNPs rs5393 and rs5394 of *SLC2A2* could be high risk genotypes to predict the conversion of T2DM in an obese Finnish subject who had impaired glucose tolerance [122]. This discrepancy is not explained at present, but it may be due to differences in the ethnic group or study population. Detailed functional studies of the effects of promoter SNPs on *SLC2A2* transcription are needed.

### 4.2. Promoter Polymorphisms in GCK Gene

Recently, a functional β*GCK* promoter mutation (−71G > C) was identified in Slovakian and British patients with GCK-MODY phenotype who have no abnormality of the *GCK* coding seqeuence. The mutation was associated with increased fasting plasma glucose (FPG) levels. In addition, the β*GCK* promoter of the −71C allele showed remarkable reductions of promoter reporter activity in INS-1 cells due to the decreased SP1 binding to −82/–67 bp region (Figure 2). These data suggested that the mutation was cosegregated with fasting hyperglycemia due to loss of SP1 binding [123].

Two SNPs, −6612G > A (rs4607517) [124,125] and −30G > A (rs1799884) [126–129], in the β*GCK* promoter were significantly associated with fasting plasma glucose level in several populations. Moreover, the −30G > A SNP has been associated with reduced β-cell function [130], impaired glucose tolerance [131] and T2DM [126]. These data suggested that rare alleles of these two SNPs may also inhibit the β*GCK* transcription. However, functional studies of these two SNPs are needed to clarify cause-effect in terms of β*GCK* transcription.

## 5. Conclusions

Although muscle or adipose tissue have been considered as the principal organs of glucose disposal, immediate handling of hyperglycemia in β-cells or liver is also important because high levels of glucose are toxic to various tissues. Thus, studies on the regulation of glucose sensors in the liver and β-cells are important in understanding T2DM and preventing the long-term complications resulting from hyperglycemia. Recent advances in the analytical technologies of genomics and proteomics make it possible to unveil the existence of transcription factors and their physical interactions with DNA or other coregulators.

Deeper understanding of the role of transcription factors involved in the gene regulation of the glucose sensors in the liver and β-cells may provide important clues in the prevention of the occurrence of hyperglycemia-related complications and development of novel therapeutic drugs combating T2DM, a world-wide epidemic.

## Figures and Tables

**Figure 3.**
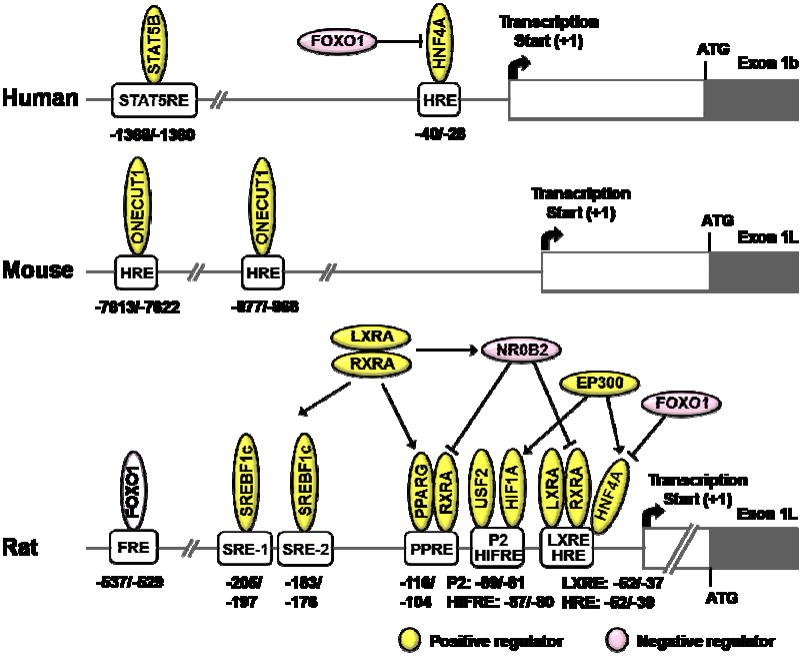
Schematics of transcriptional regulatory elements on the L*GCK* gene promoter. *Abbreviations*: STAT5B, signal transducer and activator of transcription 5B; HNF4A, hepatic nuclear factor 4 alpha; FOXO1, forkhead box O1; ONECUT1, one cut homeobox 1 (also known as HNF6); SREBF1c, sterol regulatory element binding transcription factor 1c; LXRA, liver X receptor alpha (also known as NR1H3); RXRA, retinoid X receptor alpha; NR0B2, nuclear receptor subfamily 0, group B, member 2 (also known as SHP); EP300, E1A binding protein p300; PPARG, peroxisome proliferator-activated receptor gamma; USF2, upstream stimulatory factor 2; HIF1A, hypoxia induced factor 1 alpha subunit; STAT5RE, STAT5B response element; HRE, HNF response element; FRE, FOXO1 response element; SRE, SREBF response element; PPRE, PPAR response element; HIFRE, HIF1A response element; LXRE, LXR response element.
